# SUMO E3 ligase AtMMS21 is required for normal meiosis and gametophyte development in *Arabidopsis*

**DOI:** 10.1186/1471-2229-14-153

**Published:** 2014-06-03

**Authors:** Ming Liu, Songfeng Shi, Shengchun Zhang, Panglian Xu, Jianbin Lai, Yiyang Liu, Dongke Yuan, Yaqin Wang, Jinju Du, Chengwei Yang

**Affiliations:** 1 Guangdong Key Lab of Biotechnology for Plant Development, College of Life Science, South China Normal University, Guangzhou 510631, China; 2 Vegetable Research Institute Guangdong Academy of Agriculture Sciences, Guangzhou, Guangdong 510640, China

**Keywords:** AtMMS21, SUMOylation, Gametophyte development, Meiosis, *Arabidopsis thaliana*

## Abstract

**Background:**

MMS21 is a SUMO E3 ligase that is conserved in eukaryotes, and has previously been shown to be required for DNA repair and maintenance of chromosome integrity. Loss of the *Arabidopsis* MMS21 causes defective meristems and dwarf phenotypes.

**Results:**

Here, we show a role for *AtMMS21* during gametophyte development. *AtMMS21* deficient plants are semisterile with shorter mature siliques and abortive seeds. The *mms21-1* mutant shows reduced pollen number, and viability, and germination and abnormal pollen tube growth. Embryo sac development is also compromised in the mutant. During meiosis, chromosome mis-segregation and fragmentation is observed, and the products of meiosis are frequently dyads or irregular tetrads. Several transcripts for meiotic genes related to chromosome maintenance and behavior are altered. Moreover, accumulation of SUMO-protein conjugates in the *mms21-1* pollen grains is distinct from that in wild-type.

**Conclusions:**

Thus, these results suggest that *AtMMS21* mediated SUMOylation may stabilize the expression and accumulation of meiotic proteins and affect gametophyte development.

## Background

The life cycle of flowering plants alternates between a prominent diploid sporophyte generation and a short-lived haploid gametophyte generation. The haploid gametophytes are derived from the haploid spores that are produced by diploid megasporocytes (female) and microsporocyte (male)parent cells [1]. During female gametophyte development, the megasporocyte undergoes meiosis to produce a tetrad of four haploid spores. Three of the spores degenerate, and one proceeds through three sequential rounds of mitotic division, forming the female gametophyte (embryo sac), which consists of seven cells with four cell types [2]. During male gametophyte development, microsporocytes undergo meiosis to form a tetrad of four haploid microspores. Each microspore undergoes two mitotic divisions to form the male gametophyte (pollen grain) consisting of a vegetative cell and two sperm cells [3]. Following pollination, the pollen grain lands on the pistil and extends a pollen tube that allows the delivery of the two sperm cells into the female gametophyte, and then gives rise to the diploid zygote to begin the sporophytic generation [4]. Female and male gametophyte development differ considerably, but at the same time share the same fundamental hallmark of being haploid organs: it is therefore logical that they might require the same basal machinery and share a number of common regulators [5].

Meiosis is a specialized cellular division that is conserved among most eukaryotes. This process is indispensable for formation of viable offspring. It consists of two rounds of chromosome segregation after a single round of DNA replication, giving rise to four haploid daughter cells. During meiosis I homologous chromosomes pair, undergo recombination and then segregate, whereas sister chromatids separate during meiosis II [6]. Recombination is initiated by the formation of SPO11-induced DNA double strand-breaks (DSBs), and DSBs in meiosis are repaired by homologous recombination [7]. Disruption of meiotic homologous recombination could result in chromosome anomalies, which could lead to mis-segregation and aneuploidy [8].

The faithful transmission of chromosomes during meiosis is essential for the survival and reproduction of flowering plants. A critical aspect of chromosome dynamics is structural maintenance of chromosome (SMC) proteins, which are responsible for sister chromatid cohesion, chromosome condensation and homologous recombination (HR) during meiosis [9,10]. The evolutionarily conserved SMC gene family encodes members of the three complexes: the cohesin, the condensin and the SMC5/6 complex. In *Arabidopsis*, the cohesin complex consists of the SMC1, SMC3, SCC3, and four α-kleisin subunits: SYN1, SYN2, SYN3 and SYN4 [10]. Evidence from mutants (*smc1*, *smc3*, *scc3*, *syn1*, *syn3*) defective in meiosis have shown that cohesin is essential for the control of chromosome structure and many subsequent meiotic events [11-15]. The *arabidopsis* condensin complex consists of the SMC2, SMC4, and β-kleisin subunit CAP-D2. Data from mutants (*smc2*, *smc4*) with gametophytic defects have shown that condensin is required for chromosome condensation and segregation during mitosis, meiosis and embryo development [16-18]. In plants, knowledge about the role of SMC5/6 complex is still limited. The *arabidopsis* SMC5/6 complex presumably consists of SMC5, one of two alternative SMC6 proteins and four NSE(non-SMC elements) proteins (NSE1-4) [10]. It has been shown in *Arabidopsis* that SMC5 and SMC6 enhances sister chromatid alignment after DNA damage and thereby facilitates correct DSB repair via HR between sister chromatids [19]. Although the *arabidopsis* NSE1 and SMC5 are essential for seed development [19,20], the role SMC5/6 complex in gametophyte development is still unknown.

The *Arabidopsis* SUMO E3 ligase AtMMS21/HPY2, a homologue of NSE2/MMS21, has been identified recently as participating in root development. Loss of AtMMS21/HPY2 function results in premature mitotic-to-endocycle transition, defective cytokinin signaling, and impaired cell cycle, leading to severe dwarfism with compromised meristems [21-23]. Recent data demonstrate that AtMMS21/HPY2 functions as a subunit of the SMC5/6 complex through its interaction with SMC5. AtMMS21 acts in DSB amelioration and stem cell niche maintenance during root development [24]. Hence, AtMMS21 is involved in cell division, differentiation, expansion and survival during plant development. The highly coordinated processes of cell division, differentiation, and expansion that take place during gametophyte development require precise fine-tuning of gene regulatory networks [25]. However, whether and how *AtMMS21* participates in regulating the gametophyte development and reproductive processes remains unclear.

In the present study, we provide cell-biological and molecular evidence that *AtMMS21* is required for fertility in *Arabidopsis*. Mutations in *AtMMS21* cause semi-sterility with aberrant gamete, indicating that the gene is essential for gametogenesis. Furthermore, *mms21-1* mutant cells exhibit chromosome fragmentation and mis-segregation during meiosis. Transcription level for several meiotic genes are also altered in *mms21-1* buds. These observations suggest that *AtMMS21* plays an important role in meiosis and gametophyte development.

## Results

### *mms21-1* mutant shows severely reduced fertility

**Figure 1:**
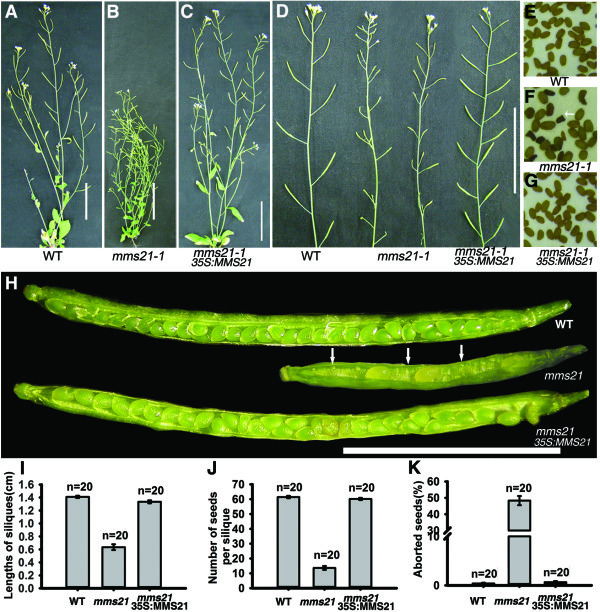
***mms21-1 *plants exhibit reduced fertility. (A-C)** Morphology of 6-week-old wild-type, *mms21-1* and *35:MMS21 mms21-1* plants under long-day growth conditions. **(D)** Primary inflorescences of wild-type, *mms21-1* and *35:MMS21 mms21-1* plants. **(E-G)** Seed phenotype in wild-type and *mms21-1* plants. **(H)** Dissected silique form wild-type , *mms21-1* and *35:MMS21 mms21-1* plants. *mms21-1* showing severly reduced seed-set and unfertilized ovules. **(I)** The lengths of siliques in wild-type , *mms21-1* and *35:MMS21 mms21-1*. **(J)** Numbers of seeds per silique in wild-type, *mms21-1* and *35:MMS21 mms21-1*. **(K)** Percentage aborted seeds per silique in wild-type , *mms21-1* and *35:MMS21 mms21-1*. Bars = 5 cm in **(A-C)**, 1 cm in **(E)**, 5 mm in **(H)**.

Previous studies showed that mutation of *AtMMS21/HPY2* resulted in severe developmental defects [21,22,24]. To determine whether AtMMS21 regulates the reproductive development, we first analyzed the fertility of *mms21-1* and wild-type plants. In their reproductive phase, *mms21-1* plants were bushy with short siliques (Figure 1A-D). Mean silique length was reduced to 6.3 ± 0.44 mm in *mms21-1*, compared with 14.1 ± 0.18 mm in the wild-type (Figure 1I). Ten days after pollination, dissected siliques from *mms21-1* plants showed severely reduced seed-set and unfertilized ovules (Figure 1H). Later in mature siliques, the mean seed-set was only 13.7 ± 1.33 per silique, accounting for 22.2% of the normal seed-set in the wild-type (Figure 1J). Some of the mutant seeds were abnormal in appearance with a dark and shrunken seed coat (Figure 1F). The percentage of aborted seeds in *mms21-1* is approximatly 48.3%, while only 0.4% in wide-type (Figure 1K). Furthermore, we analyzed fertility in the transgenic plants expressing *35S::AtMMS21-GFP* in *mms21-1*, and found that the expression of *AtMMS21-GFP* could rescue the semisterile phenotype of *mms21-1* (Figure 1C,D,G,H), indicating that the impaired fertility of the *mms21-1* is caused by the absence of *AtMMS21*. Therefore, these results suggested that AtMMS21 is essential to fertility in *Arabidopsis*.

### Decreased fertility in *mms21-1* is caused by both abnormal male and female fertility

**Figure 2:**
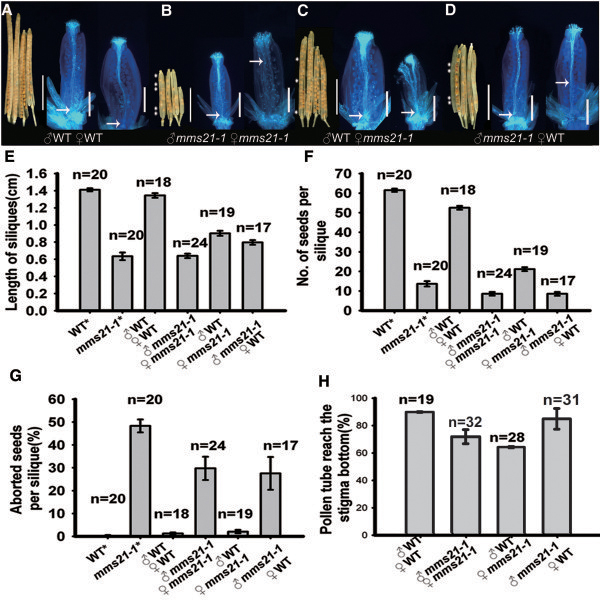
***In vivo *reciprocal cross-pollination of *mms21-1 *to wild-type plants. (A-D)** Pistils were fixed at 12 h after pollination, and pollen tube growth was examined by aniline blue staining, bars = 500 μm. The remaining pollinated pistils ripened into mature siliques in 14 d, and siliques were then dissected for examination of fertilized ovules. Arrows indicate the pollen tube front in the pistil. Asterisks designate unfertilized ovules in the silique. **(E-H)** In another set of reciprocal cross-pollinations, sizes of mature siliques, numbers of seeds per silique and percentage aborted seeds per silique were examined 14 d after pollination. Control flowers were allowed to self-pollinate (asterisks). Data are shown as mean ± SD. **(E)** Percentage of pollen tubes growing to the base of the pistil. Bars = 5 mm.

To answer the question of whether male or female fertility was affected by the mutation, we performed reciprocal cross-pollinations between *mms21-1* and wild-type plants. In reciprocal cross-pollinations, wild-type pollen showed active pollen tube growth to the base of the wild-type pistil in 12 h, and the average size of mature siliques and number of seeds per silique from this cross were equivalent to those of self-pollinated wild-type plants (Figure 2A, F, G). By contrast, *mms21-1* pollen did not show normal fertilization in either *mms21-1* or wild-type pistils. Unfertilized ovules were random distributed in the mature siliques and a high percentage of shrunken seeds (Figure 2B, D, G), and short siliques and small numbers of seeds per silique, (Figure 2E, F), indicating that the function of the pollen was compromised in the *mms21-1* mutants.

Cross-pollination of wild-type pollen onto *mms21-1* pistils resulted in better fertilization and silique sizes (Figure 2C). However, the siliques size and seed number per silique were still decreased in this cross, compared with the wild-type self-cross (Figure 2E, F). Cross-pollination of *mms21-1* pollen onto wild-type pistil showed a lower percentage in pollen tube growth to the base of the pistil (Figure 2H). Taken together, our reciprocal cross-pollination studies suggested that AtMMS21 has a function in both male and female fertility.

### *mms21-1* mutant shows reduced pollen number, viability, germination and abnormal pollen tube growth

**Figure 3:**
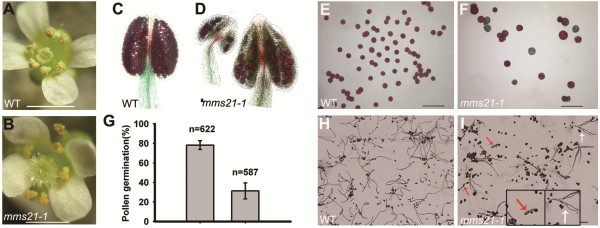
**Mutation in *AtMMS21 *affects pollen number, viability, germination and growth. (A-B)** Flower of wild-type and *mms21-1* plants. *mms21-1* produced dramatically reduced pollen grains. **(C-D)** Anthers of wild-type and *mms21-1* plants, which are stained with Alexander’s solution. **(E-F)** Pollen grains of wild-type and *mms21-1* plants, which are stained with Alexander’s solution. The red-stained cytoplasm indicates viable pollen grains, whereas nonviable pollen grains are stained blue. **(G)** Percentage of pollen germination. **(G-I)***In vitro* pollen tube growth assay. White arrows indicate long pollen tubes exhibiting branched tips, and red arrows indicate short pollen tubes tip with obviously swollen. Bars = 5 mm in **(A-B)**, 2 mm in **(E-F, H-I)**.

To further characterize the semisterile phenotype of *mms21-1* plant, we first examined the effects of the *mms21-1* mutation on male fertility. Unlike wild-type (Figure 3A), there were few pollen grains produced on the surfaces of anthers and stigma in *mms21-1* flowers (Figure 3B). 861 ± 135(n = 90) pollen grains per wild-type flower but only about 136 ± 53(n = 90) pollen grains were observed in *mms21-1*flowers. To assess pollen grain viability, anthers and mature pollen grains from both the wild-type and mutant flowers were stained with Alexander’s solution [26]. Wild-type mature anthers were in uniform size, and the pollen grains stained red, which indicates viability (Figure 3C, E). In contrast, *mms21-1* mature anthers were variable in size and shape (Figure 3D, F). Pollen grains in *mms21-1* plants were generally bigger with about 30.0% nonviable pollen grains, as indicated by blue staining, whereas the wild-type produced <1.9% abnormal pollen (Figure 3F). *In vitro* pollen germination and pollen tube growth assays were also performed. In *mms21-1*, pollen tube initiation and growth occurred in only 31.3% of the pollen grains while 78.2% of wild-type pollen germinated (Figure 3 G-I). Particularly, *mms21-1* pollen tubes showed a variable phenotypes and abnormal morphologies. For example, long pollen tubes exhibiting branched tips (red arrows in Figure 3I) and short swollen pollen tubes were observed (white arrows in Figure 3I). These results indicated that *AtMMS21* mutation affects pollen number, viability, germination and tube growth.

### Mutation of *AtMMS21* causes defects in gametogenesis

**Figure 4:**
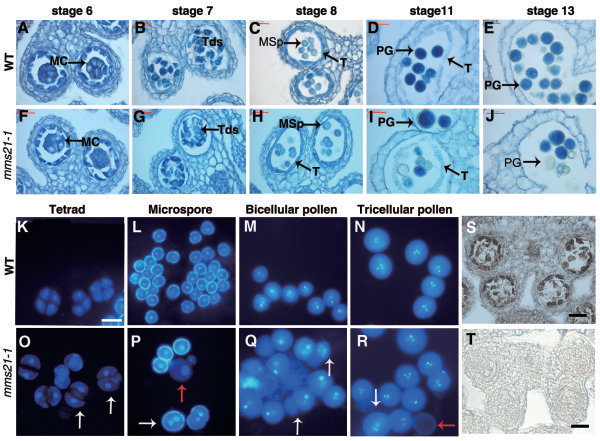
**Male gametophyte development is defective in *mms21-1 *mutants.** Anther development at stages 6, 7, 8, 11, 13 in the wild-type **(A–E)** and *mms21-1* mutant **(F–J)**. **(A, F)** Anther stage 6. PMCs have separated and are ready to undergo meiosis. **(B, G)** Anther stage 7. Uniform size tetrads (Tds) in wild-type, dyads and triads in *mms21-1* mutant. **(C, H)** Anther stage 8. Note the dyads in *mms21-1* mutant. **(D, I)** Anther stage 11. Pollen were uniform size in wild-type but variable size and shrunken in *mms21-1* mutant. **(E, J)** Anther stage13. In *mms21-1* mutant pollen grains were in variable size and shrunken.MC, meiotic cell; Tds, tetrads; MSp, microspores; PG, pollen grains; T, tapetum. Bars = 20 μm. **(K-N)** DAPI staining of wild-type anthers at different development stages. Pollen grains are of uniform size in the wild-type. **(O-R)** DAPI staining of *mms21-1* anthers at stages equivalent to **(K-N)**. Arrows in **(O)** indicate that there are dyads and triads at tetrad stage in *mms21-1*. Arrows in **(P)** indicate that visible variable size (red arrow) and some had two nuclei (white arrow) in uninucleus microspore stage in *mms21-1.* Arrows **Q** indicate that abnormal nuclear contents pollen Arrows in **R** indicate larger pollen (white arrow) and pollen with no DAPI staining (red arrow) in *mms21-1*. Bars = 20 μm. **S**, *AtMMS21* expression was detected in PMCs by in situ hybridization. **T**, No hybridization signal was detected when a sense probe was used.

To determine which step of pollen development is affected by the *mms21-1* mutation, paraffin-cross sections of anthers stained with toluidine blue from various developmental stages were analyzed (Additional file 1: Figure S1). In *Arabidopsis*, anther development can be divided into 14 well-ordered stages by morphological characteristics [27]. In *mms21-1*, the early 6 stages of pollen development appeared normal compared with wild-type (Figure 4A, F; Additional file 1: Figure S1). Alteration were first observed at tetrad stage, wild-type meioses produced four uniform spores, while the mutant produced a mixture of dyads, triads, and tetrads (Figure 4B, G, K, O; Additional file 2: Figure S2). At stage 8, microspores are typically released from the tetrads. Microspores were angular in shape in wild-type plants, whereas microspores of *mms21-1* plants appeared of various sizes (Figure 4C, H). During stage 11, wild-type microspores underwent asymmetric mitotic divisions and generated a significant pollen wall (Figure 4D). In contrast, in *mms21-1*, most of the microspores were degenerated (Figure 4I). Eventually, pollen grains of the wild-type were released when anther dehiscenced, whereas most of the mutant microspores were degenerated, leaving an empty locule (Figure 4E-J).

To more precisely define the developmental defect in *mms21-1* pollen, developing *mms21-1* pollen were stained with DAPI and observed at different developmental stages. The *mms21-1* meiotic products were a mixture of dyads, triads and tetrads, while the WT tetrads had four equal-sized spores enclosed in a callose wall (Figure 4K, O). At the uninucleate stage, some abnormal microspores in *mms21-1* exhibited no fluorescence or contained two nuclei within one exine wall (Figure 4P). In the bicellular pollen stage, some of the *mms21-1* pollen became shrunken and lacked DAPI staining compared with wild-type (Figure 4M, Q). The size difference became more pronounced at the tricellular stage, as normal pollen continued to approach maturity, while mutants were losing nuclear content and becoming distorted (Figure 4R). We also examined expression patten of *AtMMS21* by in situ hybridization. Transverse sections of anthers showed strong hybridization signals in pollen mother cells (PMCs; Figure 4S); no signals were detected when a sense probe was used (Figure 4T). Loss of *AtMMS21* function also causes defects in female gametogenesis. Normally the diploid megaspore mother cell undergoes meiosis and gives rise to four haploid nuclei. Subsequently, three megaspores undergo cell death, with the remaining, functional megaspore proceeding into megagametogenesis. Examination of cleared ovules in *mms21-1* mutant plants showed that some megaspore mother cells appeared to abort either before or during meiosis , giving rise to embryo sacs containing one to five nuclei (Additional file 3: Figure S3). These data indicated that AtMMS21 is important for gametogenesis, both during male and female gametophyte development.

### Disruption of *AtMMS21* leads to defects in chromosome behavior during meiosis

**Figure 5:**
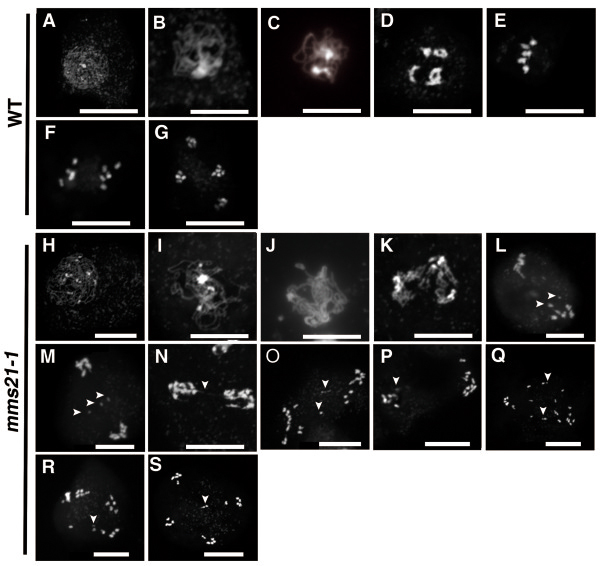
**The *mms21-1 *plants exhibit defects during meiosis.** DAPI staining of meiosis stages from wild-type pollen mother cells. Various stages of meiotic chromosome spreads from wild-type **(A–G)** and *mms21–1***(H-S)** are illustrated **(A, H)** Leptotene stage. **(B, I)** Zygotene stage. **(C, J)** Pachytene stage. **(D, K)** Diakinesis stage. **(E)** Metaphase I. **(F, L-N)** Anaphase I. **(G, O-S)** Telophase II. Arrows indicate abnormal chromosomal fragments **(I, M, O-R)**, chromosome bridges **(N)**, and lagging chromosome **(S)**. Bars = 10 μm.

The defects in gametophyte development described above could arise from defective meiosis. Therefore we examined chromosome spreads from various stages of meiosis of wild-type and *mms21-1* anthers. In wild-type meiosis, chromosomes were clearly single and unpaired in leptotene (Figure 5A) and underwent synapsis between homologous chromosomes at the zygotene stage (Figure 5B) until its completion in pachytene (Figure 5C). At diakinesis stage, homologous chromosomes desynapsed and then underwent further condensation to form five bivalents (Figure 5D). The five bivalents aligned at the division plane at metaphase I (Figure 5E). At anaphase I homologous chromosomes separated from each other and moved to opposite poles (Figure 5F). Subsequently tetrads formed at anaphase II (Figure 5G). In *mm21-1*mutant plants, the early development stages of meiotic nuclei, in other words from leptotene to pachytene, appeared to proceed normally (Figure 5H-J). Alterations were observed at diakinesis, in diakinesis, chromosomes were further condensed, but in *mms21-1* it appeared to be less condensed than wild type (Figure 5K). Interestingly, we did not observe distinct metaphase I in the *mms21–1*. Furthermore, chromosome fragments and bridges between bivalents were observed in anaphase I (Figure 5L-N) In anaphase II, segregation of the sister chromotids were also disturbed leading to irregular meiotic products with variable DNA contents (Figure 5O-Q). As some lagging chromosome dispersed throughout the cytoplasm, typical tetrads were rarely found (Figure 5R-S). These observations showed that loss function of *AtMMS21* cause abnormal meiotic chromosome behavior.

### *mms21-1* mutants exhibits altered transcript levels for meiotic genes

**Figure 6:**
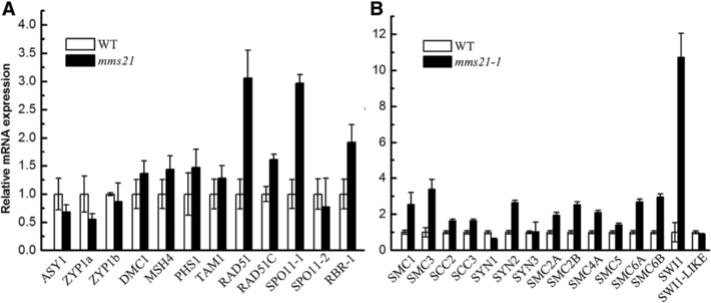
**Expression analyses of meiotic and structural maintenance of chromosome genes in wild-type and *mms21-1 *flowers.** Quantitative PCR was used to measure transcript levels of meiotic genes in buds RNA isolated from wild-type and *mms21-1* mutant plants. **(A)** Expression analysis of select synapsis, recombination in wild-type and *mms21-1* plants. **(B)** Expression analysis of select cohesin, condensin, SMC5/6 complex and SMC-like genes in wild-type and *mms21-1* plants. Data presented here represent three biological replicate experiments. Each quantitative RT-PCR was repeated at least three times on each biological replicate. Bars are averages ± SE.

Meiocytes exhibit abnormal chromosome behavior, we investigated whether loss of *AtMMS21* affected meiotic gene expression, which includes *ASY1*[28], *ZYP1*[29], *DMC1*[30], *TAM*[31], *MSH4*[32], *PHS1*[33], *RAD51*[34], *RAD51C*[35]*, RBR1*[36], *SPO11-1*[37], and *SPO11-2*[38] by the qRT-PCR. Although transcript levels for *DMC1*, *MSH4*, *SPO11-2* and *TAM1* were similar in the buds of wild-type (Figure 6A), many meiotic gene expression were changed in the flower buds of *mms21-1*. For example, transcripts for *SPO11-1* and *RAD51* were found to be increased approximately 3 and 3.5 fold, respectively. *RBR* is essential for inter-homologue recombination and synapsis [36], and transcripts for RBR were up-regulated in *mms21-1* mutant. *ZYP1* encodes a synaptonemal complex protein and *ASY1* encods an axis-associated protein in *Arabidopsis*[28,29]; *ZYP1a* and *ASY1* transcript levels were down-regulated compared with wild-type plants. In addition, transcripts of cohesin, condensin, SMC5/6 complex and SMC-like genes in *mms21-1* plants were elevated, with the exception of *SYN1* and *SWI1-like* (Figure 6B). Particularly, the transcript abundance level of *SWI1* was dramatically increased.

### *mms21-1* mutant exhibits reduced SUMOylation levels in pollen grains

**Figure 7:**
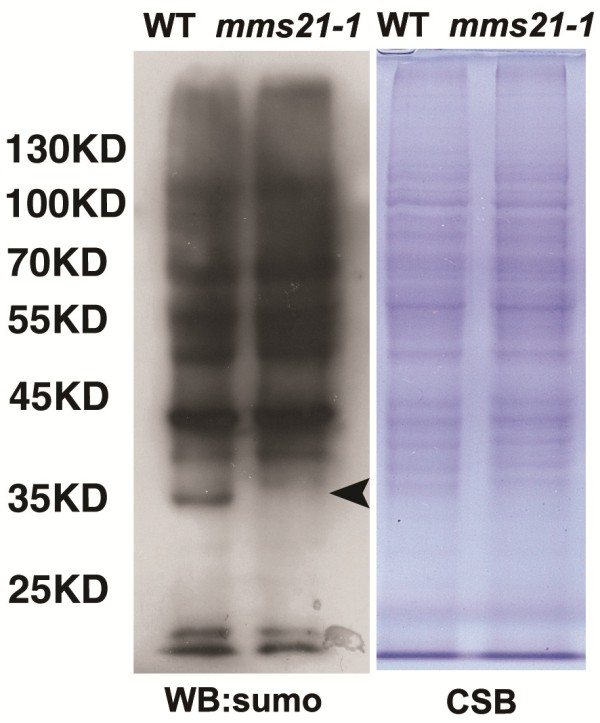
**SUMOylation profiles of pollen proteins in wild-type and mms21-1 plants.** Pollen protein extracts were analyzed by protein gel blots using anti-SUMO1polyclonal antibodies to detect SUMO-protein of wild-type and *mms21-1*. Arrows indicate missing SUMO conjugates in *mms21-1*. Coomassie brilliant blue staining of total protein was used as the loading control.

Because *AtMMS21* encodes a SUMO E3 ligase and mutation of AtMMS21 causes defective gametocyte, it is reasonable to assume that accumulation of SUMO-protein conjugates in generative cell were altered in *mms21-1* mutant. An immunoblot of total pollen grain proteins by anti-AtSUMO1 demonstrated a difference in the SUMO conjugates in wild-type and *mms21-1*, as some SUMOylation conjugates were missing in *mms21-1* pollen grains proteins (Figure 7). These results suggested that AtMMS21 is involved in the SUMOylation of *Arabidopsis* pollen grains proteins, and may regulate the gametophyte developmental process through the SUMOylation pathway.

## Discussion

SUMOylation and the components of the SUMO conjugation machinery are essential for viability, as shown by the embryo lethality of the mutations in *SAE2* or *SCE* or both in *SUMO1* and *SUMO2*[39]. However, the role of SUMOylation for gametophyte development is poorly understood because of the zygotic lethality. Here, we used a viable mutant without functional SUMO E3 ligase AtMMS21 for studying the function of SUMOylation in reproductive development. First, we examined T-DNA mutants of *AtMMS21*[21], focusing on silique size and seed set, which are direct indicators of successful fertility. The *mms21-1* mutant exhibits a drastic reduction in fertility, embodied by a shorter silique length with a smaller seed set than those in the wild-type (Figure 1). Examination of the male fertility indicated that mutant anthers produce fewer functional pollen grains (Figure 3). Cytological observation of various developmental stages in *mms21-1* pollen revealed that the products of meiosis in the mutant were mostly dyads of spores instead of tetrads (Figure 4). Similarly, mutant meiosis produced abnormal embryo sacs during female gametogenesis (Figure 2). These results are consistent with our reciprocal cross study suggesting that AtMMS21 has crucial roles in both male and female gametogenesis.

Gametogenesis is an essential process for plant reproduction and the roles of the ubiquitin system in different processes during gametogenesis have been studied extensively [1]. However, although ubiquitin-like proteins SUMO have emerged as a key regulator of plant development and stress response [40], there are currently little data supporting a specific role of the SUMO system in the control of reproduction. Our data presented a regulatory framework for the action of AtMMS21-dependent SUMOylation in reproductive development. The loss of function mutant *mms21-1* is still viable probably due to the action of another SUMO E3 ligase SIZ1 [41]. The *siz1-2* and *mms21/hpy2* double mutant results in embryonic lethality, supporting the notion that AtMMS21 and SIZ1 have overlapping functions [42]. Mature female gametophytes were rapidly disrupted in the absence of the SIZ1 protein, while pollen developed well, indicating that SIZ1 plays important roles in sustaining the stability of the mature female gametophyte [42]. However, unlike the SIZ1, AtMMS21 is involved in the development and the female and male gametophyte. Interestingly, AtMMS21 was recently shown to be expressed highly in reproductive organs such as anther using a GUS reporter construct [42]. The expression data supported the role of AtMMS21 during gametophyte development inferred from the *mms21-1* mutant lines. The severe defects in *mms21-1* gametophyte indicated that AtMMS21 is vital for fundamental processes (e.g. meiosis), thereby ensuring normal reproductive development in *Arabidopsis*.

The precise transmission of chromosomes from mother to daughter cells is a highly controlled process that requires members of the SMC (structural maintenance of chromosome) protein family. Although cohesin (SMC1/3) and condensin (SMC2/4) complexes have been reported to be involved in many aspects of meiosis, the role of the SMC5/6 complex in meiosis remains elusive. Here, we demonstrated that a SMC5/6 subunit, AtMMS21, is essential during *Arabidopsis* gametogenesis. The lack of AtMMS21 resulted in severely disrupted meiosis with bridges between chromosomes and chromosome fragmentations during meiosis I, leading to the unequal distribution of meiotic products and polyad formation during meiosis II (Figure 5). AtMMS21 is an important regulator of cell cycle progression [23,24]. It is possible that the dyad cells may result from delayed progression of meiosis. During meiosis, a germ cell will purposely create double-strand breaks which is induced by the protein SPO11 to promote chiasmata formation, and the programmed DSBs which are repaired by HR [43]. If the DSBs remain unrepaired while the cell cycle continues, it will possibly lead to fragmentation and mis-segregation [44]. Recent study demonstrated that AtMMS21, a SMC5/6 complex sub-unit, is involved in DSB repair [26]. Chromosome fragmentation and mis-segregation observed in meiosis of *mms21-1* may due to defective DSB repair. The chromosome fragmentation may result from failure of DSB repair or non-condensed entangled chromosomes pulled to break by the spindle. This notion is consistent with previous finding that the yeast SMC5/6 complex is required to ensure proper chromosome behavior during meiosis [45,46]. In addition, several genes associated with DSB accumulation or formation, such as *RAD51 RAD51C* and *SPO11-1* were increased in *mms21-1* flower buds (Figure 6A), suggesting that *mms21-1* generative cells may contain unrepaired DSBs. Therefore, it will be interesting to examine whether AtMMS21 is recruited to mitotic DSBs and function in meiotic recombination during meiosis. Although the meiotic functions of AtMMS21 need to be investigated, our data demonstrated that AtMMS21 is required for proper chromosome behavior and successful meiotic divisions.

Meiotic roles have been discovered for cohesins and condensins in plants [10]. Here we show that a SMC5/6 associated protein AtMMS21 is important for meiosis. Furthermore, several genes encode various components of the different SMC complexes in *mms21-1* plants display increased transcription level (Figure 7B). SMC complexes and their associated proteins are essential for sister chromatid cohesion, chromosome condensation, DNA repair and recombination. It is tempting to speculate that *AtMMS21* gene may affect meiotic process indirectly by altering the expression of SMC complexes and their associated genes. When exposured to DNA damaging agents all subunits of cohesin become SUMOylated, such as the SUMOylation of SCC1 is carried out by the SUMO E3 ligase MMS21 in yeast [47]. Therefore, further analysis of the protein expression of SMC complexes in the *mms21-1* meiocytes and the relation between AtMMS21 and cohesin/condensin complexes will help to clarify the precise function of AtMMS21 during meiosis. At the protein level, our results demonstrated that SUMOylation level in *mms21-1* pollen grains proteins is different from the wild-type (Figure 7), indicating that AtMMS21-mediated SUMOylation may participate in generative cell formation. Identification and functional analyses of sumoylated proteins related to AtMMS21 are necessary for the understanding how AtMMS21-dependent SUMOylation participates in meiosis and gametophyte development.

## Conclusions

In conclusion, our studies found that *Arabidopsis* MMS21 is important for gametogenesis, both during male and female gametophyte development. The loss of the *Arabidopsis* MMS21 causes reduced pollen number, viability, germination and abnormal meiotic chromosome behavior. Several transcripts for meiotic genes related to chromosome maintenance and behavior are altered in the *mms21-1* plant. Futhermore, SUMO-protein conjugates in the *mms21-1* pollen grains are different from those in wild-type. Thus, these results indicated that AtMMS21 mediated SUMOylation may stabilize the expression and accumulation of meiotic proteins in the gametophyte development.

## Methods

### Plant materials and growth conditions

The *mms21-1* mutant and 35S:AtMMS21 Arabidopsis (*Arabidopsis thaliana*; Columbia-0 ecotype) were isolated as described previously [21]. *Arabidopsis* plants were grown in a controlled growth room at 22 ± 2°C under long-day conditions (16-h light/8-h dark). For in vitro experiments, seeds were surface-sterilized for 2 min in 75% ethanol, followed by 5 min in 1% NaClO solution and washed five times in sterile distilled water, plated on growth medium (MS medium, 1.5% sucrose and 0.8% agar), vernalized at 4°C for 2 days in the dark and then exposed to white light.

### In vitro pollen tube growth assay

Plants were removed from the growth chamber for 2 h before pollen was removed from flowers. Pollen was grown on solid germination medium (0.01% boric acid, 5 mM CaCl_2_, 5 mM KCl,1 mM MgSO_4_, 10% sucrose pH 7.5, 1.5% low-melting agarose) [48] at room temperature in dark. Pollen tube length and tip morphology were examined at various time points (2 to 16 h) using a Leica dissecting microscope for higher magnification. The relative length of pollen tubes was measured at 12 h using the tool DIGIMIZER 3.2.1.0.

### Study of in vivo pollen tube growth and seed formation in siliques

To examine in vivo pollen tube growth, about 10 mature flowers at stage 14 [49] were fixed in acetic acid/ethanol (1:3) solution. Fixed floral tissues were cleared in 4 M NaOH and stained with aniline blue following a previously published method [50]. Pollen tube growth in the pistil was examined using a fluorescent compound microscope (Leica microscope DM2500).

To evaluate fertilization, mature siliques were measured for their lengths and dissected to identify aborted seeds. Siliques were also decolorized by incubation in 100% ethanol at 37°C overnight to visualize the seed set.

For in vivo reciprocal cross-pollination, 40 floral buds at stage 12 [49] were emasculated per cross a day before hand-pollination. Fresh pollen at flower stage 13 [49] was fully applied to the stigma of the emasculated flower. After a 12-h pollination, the pollinated pistil was fixed with 25% acetic acid in ethanol, hydrated with an ethanol series (70%, 50% and 30% ethanol), and treated with 8 M NaOH overnight to allow softening, the pollen tube distribution in each silique was observed after staining with 0.1% (w/v) aniline blue solution containing 108 mM K_3_PO_4_ at pH 11 and 2% glycerol, and examined as described above.

For the fertilization study, half of the pollinated flowers were further grown in the growth chamber for 8 to 10 d. Siliques were dissected or decolorized at maturity to examine seed set.

### Microscopic investigations of anther development after paraffin-section

Anthers were fixed in FAA (10% formaldehyde, 3% acetic acid and 43.5% ethanol) placed under vacuum for 1 h and then keep room temperature. After dehydration in a graded ethanol series and diaphaneity in clearing medium, the material was embedded in Paraffins (from HuaShenPai). Sections (6 μm) were obtained with a Leica Reichert Supernova microtome, placed on glass slides, and stained with a solution of 1%(w/v) toluidine blue O (toluidine blue O 1 g, 95% alcohol 4 mL, 10% acetic acid 10 mL, distilled water 86 mL). Sections examined using a Leica fluorescent compound microscope and Images were captured with a Leica DFC420 camera, and processed with Leica Application Suite software.

The images of whole morphology of WT and mutant were captured using SONY DSC-H50 camera. And the flowers, siliques and seeds morphology were examined using a Leica dissecting microscope.

### Pollen grain viability assay

Anthers were removed from flowers and mounted in a drop of Alexander’s [26] stain under a cover glass to study the abundance of pollen grains, and mature pollen grains from the WT and mutant flower buds and stained with Alexander’s staining solution, and examined using a fluorescent compound microscope (Leica microscope).

### Light and fluorescence microscopy

The DAPI (4,6-diamino-2-phenylindole dihydrochloride) staining of chromosomes in the male meiocytes was performed according to the method reported by Ross *et al*. [51]. The young buds were fixed with Carnoy’s fixative. Fixed buds were rinsed in five changes of 1 min washes in acetic buffer (10 mM sodium acetic, pH 4.5). Buds were digested with 0.3% cytohelicase, 0.3% cellulose and 0.3% pectolyase in distilled water for an hour at 37°C, and then washed with 10 mM acetic buffer two times. A drop of 60% acetic acid was added to the glass slide and the slide was incubated at 42°C for 1–2 min. Slides were stained with 2 mg/mL of DAPI (Sigma-Aldrich) and examined by a Leica DM2500 fluorescence microscope.

For the analysis of spores at earlier stages, single anthers were dissected from isolated buds using a dissecting microscope (Zeiss, Stemi SV8).Anthers were disrupted on microscope slides using dissecting needles and gently squashed in DAPI staining solution (0.8 μg/mL) under a coverslip.

To determine at which developmental stage the mutant defection, inflorescences containing buds at different developmental stages were fixed in ethanol: acetic acid (3:1; v/v) and stored at 4°C. Buds were dissected on a microscope slide and microspores or pollen were stained in 0.8 μg/mL DAPI.

Differential interference contrast microscopy was used to observe female gametophytes that had been fixed in ethanol: acetic acid (3:1) and cleared using chloral hydrate solution (8 g of chloral hydrate, 1 mL of glycerol, and 2 mL of water). Images were captured with Leica DM2500 microscope.

### Expression analysis

Total RNA was extracted from buds with the TRizol (Invitrogen), and 10 μg of RNA was treated with DNase I (TAKARA, http://www.takara-bio.com) and used for cDNA synthesis with an oligo (dt) primer and a First Strand cDNA Synthesis Kit (TAKARA). PCR was performed with the SYBR-Green PCR Mastermix (TAKARA) and amplification was monitored on a MJR Opticon Continuous Fluorescence Detection System (Bio-Rad). At least three biological replicates were performed, with three technical replicates for each sample. The sequences of primers used in these studies are presented in Additional file 4: Table S1.

The DIG RNA Labeling Kit (Roche) and DIG ProbeSynthesis Kit (Roche) were used for the *in situ* hybridization. An AtMMS21-specific cDNA fragment of 297 bp was amplified and cloned into the pSKvector. Antisense and sense digoxigenin-labeled probes were prepared as described by Zhu et al. [52]. The primers for the *in situ* hybridization were hmms21F (5′—GGATCC ATTCTGTGGCTGAGTTATTG-3′) and mms21R (5′-CTGCAG ATGTCATGTTTAGAAGAGGG-3′).

### Analysis of SUMOylation profiles

Total protein of mature pollen grains were extracted with extraction buffer (50 mM PBS PH = 7.4, 200 mM NaCl, 10 mM MgCl_2_, Glycerol 10%, add protease inhibitor cocktail tablets 10 mL/one mini tablet, Roche) and separated by SDS–PAGE. Proteins were separated on a 10% polyacrylamide gel and transferred to PVDF membrane. Polyclonal SUMO1 antibody (Agrisera) diluted in the ratio 1:3000 was applied followed by an anti-rabbit IgG coupled to HRP and detected using ECL plus (Amersham Pharmacia).

## Competing interests

The authors declare that they have no competing interests.

## Authors’ contributions

ML, SFS, SCZ, PLX, CWY analyzed and interpreted the data, and participated in manuscript preparation. YYL, DKY did the light and fluorescence microscopy. JL, YQW and JD helped with data analysis. CWY participated in the design and interpretation of experiments. All authors read and approved the final version of the manuscript.

## Supplementary Material

Additional file 1: Figure S1 Anther development at stages 3 and 5 in the wild-type and *mms21-1*. Early stages of pollen development in *mms21-1* were comparable to wild-type. Anther stage 3 (A, C): cell division events occurred within the developing anther primordial. Anther stage 5 (B, D): the typical four-lobed anther morphology is established, and PMCs have formed in the center of each lobe. E, epidermis; MMC, microspore mother cell; Sp, sporogenou; T, tapetum. Bars = 20 μm. Click here for file

Additional file 2: Figure S2 Quantitative analysis of the number of irregular meiotic products in *mms21-1* mutants. (A) Wild-type tetrads. (B-E) Irregular meiotic products in *mms21-1* mutants. B,Monad. C, Dyad. D,Triad. E,Irregular tetrads. (F) Quantitative analysis of the number of irregular meiotic products in *mms21-1* mutants. 354 *mms21-1* meiotic products observed, 120 were normal tetrads(33.9%). 103 were irregular tetrads (29.1%), 36 triad (10.2%), 78 dyad(22.0%), 17 monad(4.8%). Click here for file

Additional file 3: Figure S3 Female gametophyte development is disrupted in *mms21-1* mutants. (A) Mature embryo sac from wild-type plants. The positions of antipodal cells (white star), egg cell (black arrowheads), central cell nuclei (white arrowheads), and synergids (white star) are indicated. (B-D) Abnormal mature embryo sac from *mms21-1* mutant plants. Embryo sacs containing one (B), two (C), or five (D) nuclei were observed in ovules in the *mms21-1* mutants. Bars = 10 μm. Click here for file

Additional file 4: Table S1 Primers used in this study. Click here for file
