# Supplementary figures and images for: SUMO E3 ligase AtMMS21 is required for normal meiosis and gametophyte development in Arabidopsis
Source: BMC Plant Biol. 2014 Jun 3;14:153. doi: 10.1186/1471-2229-14-153 (PMC4189105; doi:10.1186/1471-2229-14-153)

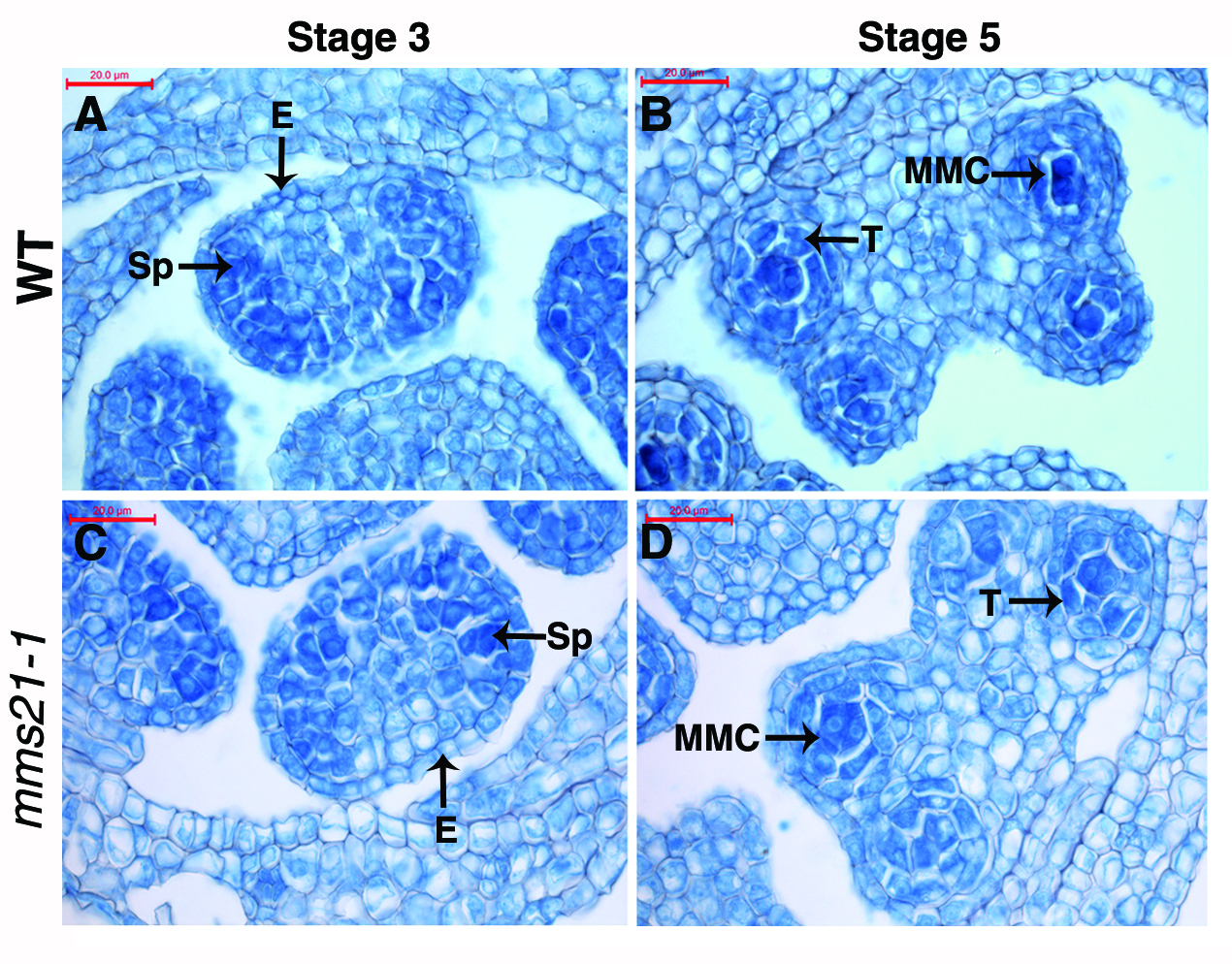

Supplement: Additional file 1: Figure S1 — Anther development at stages 3 and 5 in the wild-type and mms21-1. Early stages of pollen development in mms21-1 were comparable to wild-type. Anther stage 3 (A, C): cell division events occurred within the developing anther primordial. Anther stage 5 (B, D): the typical four-lobed anther morphology is established, and PMCs have formed in the center of each lobe. E, epidermis; MMC, microspore mother cell; Sp, sporogenou; T, tapetum. Bars = 20 μm. [file 1471-2229-14-153-S1.jpeg]

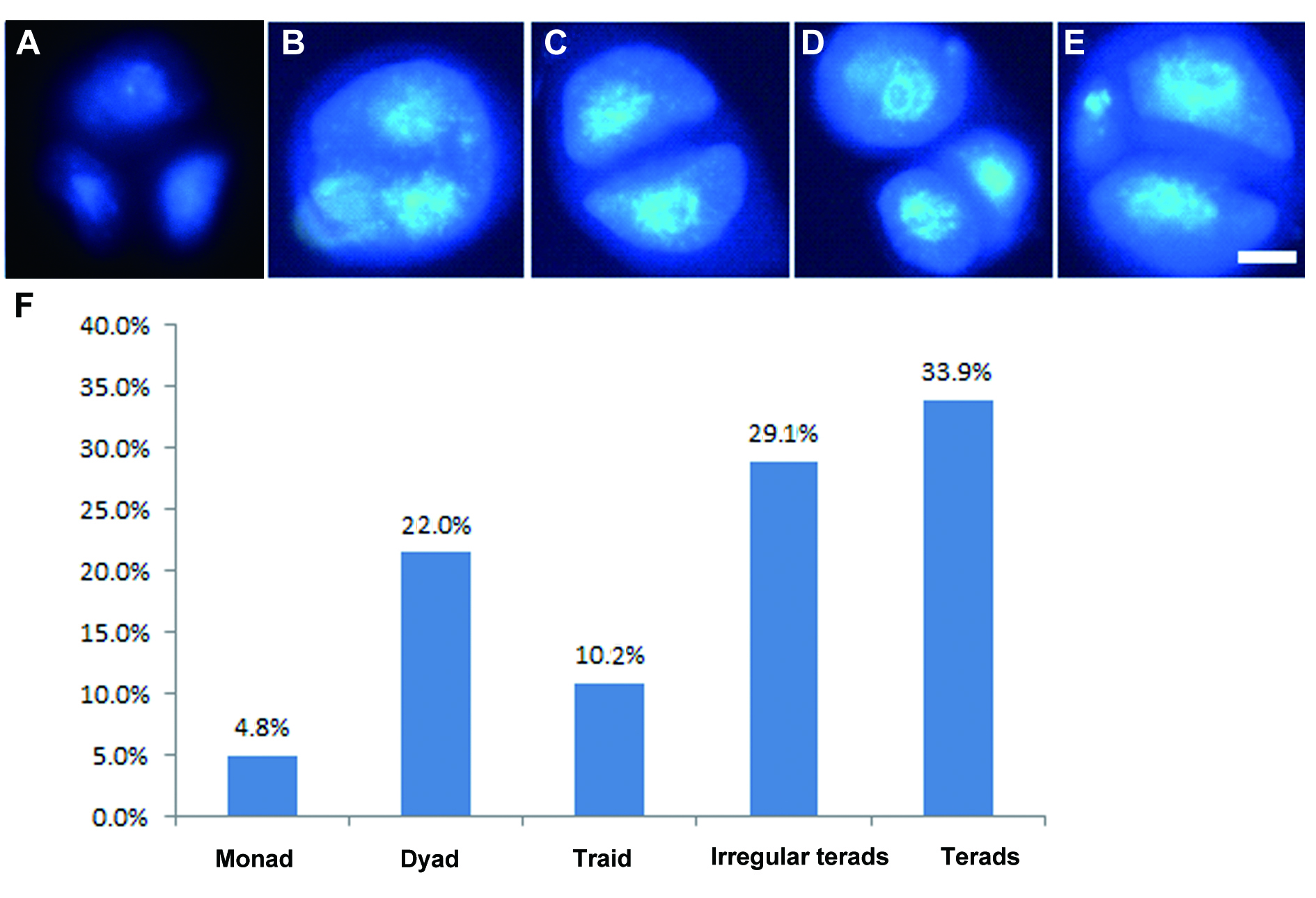

Supplement: Additional file 2: Figure S2 — Quantitative analysis of the number of irregular meiotic products in mms21-1 mutants. (A) Wild-type tetrads. (B-E) Irregular meiotic products in mms21-1 mutants. B,Monad. C, Dyad. D,Triad. E,Irregular tetrads. (F) Quantitative analysis of the number of irregular meiotic products in mms21-1 mutants. 354 mms21-1 meiotic products observed, 120 were normal tetrads(33.9%). 103 were irregular tetrads (29.1%), 36 triad (10.2%), 78 dyad(22.0%), 17 monad(4.8%). [file 1471-2229-14-153-S2.jpeg]

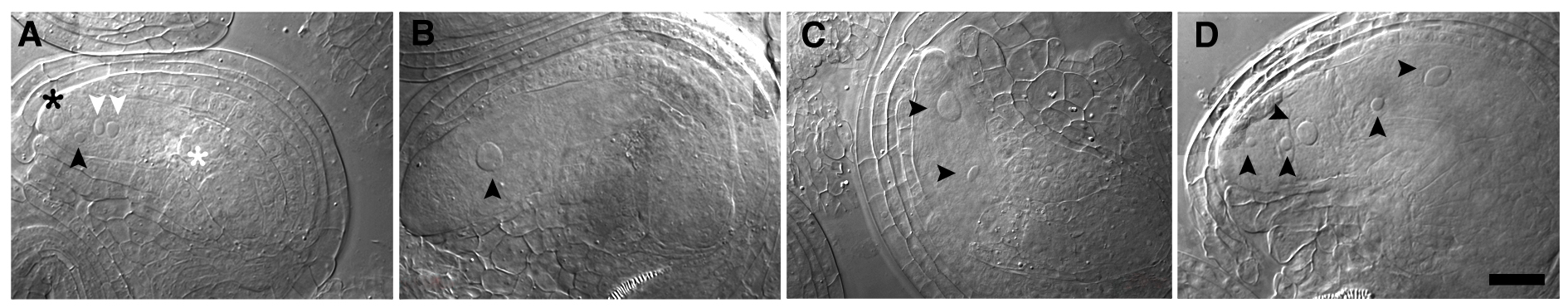

Supplement: Additional file 3: Figure S3 — Female gametophyte development is disrupted in mms21-1 mutants. (A) Mature embryo sac from wild-type plants. The positions of antipodal cells (white star), egg cell (black arrowheads), central cell nuclei (white arrowheads), and synergids (white star) are indicated. (B-D) Abnormal mature embryo sac from mms21-1 mutant plants. Embryo sacs containing one (B), two (C), or five (D) nuclei were observed in ovules in the mms21-1 mutants. Bars = 10 μm. [file 1471-2229-14-153-S3.jpeg]
