# Supplementary material for: SUMO E3 ligase AtMMS21 is required for normal meiosis and gametophyte development in Arabidopsis
Source: BMC Plant Biol. 2014 Jun 3;14:153. doi: 10.1186/1471-2229-14-153 (PMC4189105; doi:10.1186/1471-2229-14-153)
Supplement: Additional file 4: Table S1 — Primers used in this study. [file 1471-2229-14-153-S4.doc]

**Table S1 Primers used in this study.**

| *primer name* | *Sequence (5’ to 3 ’)* | *Purpose* |
| --- | --- | --- |
| *PHSl-F* | CATTGTCGACGAATTGAACC | qRT-PCR |
| *PHSl-R* | CATCTGAGGCCATGTGAAGT | qRT-PCR |
| *MLHl-F* | AACCCAAGCCACAAAGTCTC | qRT-PCR |
| *MLHl-R* | CAGCTGTCAACTCCAGCAAT | qRT-PCR |
| *SPOll-l-F* | ATGCATCCATCAGCTTTCAA | qRT-PCR |
| *SPOll-l-R* | TTTAACCAACCCATCACCAA | qRT-PCR |
| *SPOI1-2-F* | CCACAAGGTTTTTCCTCCAC | qRT-PCR |
| *SPOll-2-R* | CAAGTCCCATCCCTATGCTT | qRT-PCR |
| *ZYPlb-F* | AAGAGCTTTGGTGCAGTTAC | qRT-PCR |
| *ZYPlb-R* | CACTTTGACCGACGAATGTG | qRT-PCR |
| *ZYPla-F* | GAGTGAGGTCTGACAATGAT | qRT-PCR |
| *ZYPla-R* | CTGCGTTTACATTTTGAGCT | qRT-PCR |
| *DMCl-F* | GTTCATATCAGACCCAAAAAAGCC | qRT-PCR |
| *DMCl-R* | AGATTCGGAGCATCGTAGACTTTG | qRT-PCR |
| *RAD51-F* | CTTAGGGATGCTGGTCTCTGTAC | qRT-PCR |
| *RAD51-R* | GTCAACCTTGGCATCACTAATTC | qRT-PCR |
| *RBR-F* | GGAATGGTGGAAAGACTACAACT | qRT-PCR |
| *RBR-R* | GAAAGTcAGGCTcATTTGGGA | qRT-PCR |
| *MSH4-F* | CTCCcTTTCAACAAcAGGCA | qRT-PCR |
| *MSH4-R* | GCGGcAGAcTTATTCcTGACA | qRT-PCR |
| *ASYl-F* | GAGCGTGcTTGATCcTTGTGA | qRT-PCR |
| *ASYl-R* | CAACCTCTCCATCATCGTCATCT | qRT-PCR |
| *ACTIN2-F* | AACATCCTATTCTACTTACC | qRT-PCR |
| *ACTIN2-R* | CTGAATAGCCACATACATAG | qRT-PCR |
| *AtRAD51C-F* | TCAACTAGCGCTTGCTTTAGGC | qRT-PCR |
| *AtRAD52C-R* | TGCGTAACGCTCATCACCATTC | qRT-PCR |
| *SMC2B-F* | TGAAAGTGCGCCAACTGAAGAAG | qRT-PCR |
| *SMC3B-R* | CGTGCATCTCAAGTTCCTTTGTCG | qRT-PCR |
| *SMC2A-F* | TGGCATTGCTTCTCTTCAAACCG | qRT-PCR |
| *SMC3A-R* | TCAGCGAAACCACGATAAACTGC | qRT-PCR |
| *SCC3-F* | GCTTCACCTTCTTGGAAACCTTGG | qRT-PCR |
| *SCC4-R* | TTGCTGCTTCCTTGTCGTCTTG | qRT-PCR |
| *SMC6A-F* | AGAGGGAGGCAGAAATGAATCTCG | qRT-PCR |
| *SMC6A-R* | TCTAGTTGGGTGCGTTGCTTCTTC | qRT-PCR |
| *SMC6B-F* | GAACAGAACGAGCACCACAAGC | qRT-PCR |
| *SMC6B-R* | TCCAGCGTGGAGACAGTATCAG | qRT-PCR |
| *SMC4A-F* | TCTTGACTCAATCGCCGAGTACC | qRT-PCR |
| *SMC5A-R* | AAGTTCGTCTACCCGTCCGTTG | qRT-PCR |
| *SMC1-F* | CGTTGCTCTTCTCCATACATAGGC | qRT-PCR |
| *SMC1-R* | CAAGCGCAGCATCCACTTCATC | qRT-PCR |
| *SYN1-F* | TCGTAGGGACGGATTTGCTGAG | qRT-PCR |
| *SYN1-R* | TGGTTGTGGTCTATCGTGTTCCTC | qRT-PCR |
| *SYN2-F* | AGTCAGGAAACAGAGCGCATGG | qRT-PCR |
| *SYN2-R* | GAAGGTCCTCTTCCATGTCAAACC | qRT-PCR |
| *SYN3-F* | TGCTTTGACAGGGAGAGCAAGAG | qRT-PCR |
| *SYN3-R* | TGTGGTGGGACTACTTGAAGAACG | qRT-PCR |
| *SCC2-F* | AAATGATGACCGGTGCCAAGCC | qRT-PCR |
| *SCC2-R* | AGGCAACGCTTTGTCTGGAGAG | qRT-PCR |
| *SMC5-F* | TCAACCAAACACACCACAGTGC | qRT-PCR |
| *SMC5-R* | TGAAGGCTCCGCGATATAAGGAC | qRT-PCR |
| *SWI1like-F* | ACTCCGGGTTCTTCGATTACTGC | qRT-PCR |
| *SWI2like-R* | CCAACTCCTTCAAGGTTTCCTGAG | qRT-PCR |
| *SWI1-F* | TCGATTAGAGTCGTCATGGTGAGC | qRT-PCR |
| *SWI2-R* | AACATGCTTGGGTACCGGAGAG | qRT-PCR |
